# Structure-Function Analysis of the Glioma Targeting NFL-TBS.40-63 Peptide Corresponding to the Tubulin-Binding Site on the Light Neurofilament Subunit

**DOI:** 10.1371/journal.pone.0049436

**Published:** 2012-11-09

**Authors:** Raphael Berges, Julien Balzeau, Masayuki Takahashi, Chantal Prevost, Joel Eyer

**Affiliations:** Laboratoire de Neurobiologie & Transgenèse, UPRES EA 3143, INSERM, Centre Hospitalier Universitaire, Angers, France; NIH/NCI, United States of America

## Abstract

We previously reported that a 24 amino acid peptide (NFL-TBS.40-63) corresponding to the tubulin-binding site located on the light neurofilament subunit, selectively enters in glioblastoma cells where it disrupts their microtubule network and inhibits their proliferation. Here, we analyzed the structure-function relationships using an alanine-scanning strategy, in order to identify residues essential for these biological activities. We showed that the majority of modified peptides present a decreased or total loss to penetrate in these cells, or to alter microtubules. Correspondingly, circular dichroism measurements showed that this peptide forms either β-sheet or α-helix structures according to the solvent and that alanine substitution modified or destabilized the structure, in relation with changes in the biological activities. Moreover, substitution of serine residues by phosphoserine or aspartic acid concomitantly decreased the cell penetrating activity and the structure stability. These results indicate the importance of structure for the activities, including selectivity to glioblastoma cells of this peptide, and its regulation by phosphorylation.

## Introduction

Neurofilaments (NFs) are the major component of the cytoskeleton in mature neurons. They are composed of three polypeptide subunits of light (NFL, 61 kDa in humans), medium (NFM, 90 kDa), and heavy (NFH, 110 kDa) molecular weight [1]. Each subunit contains a central α-helical rod domain of ∼310 residues, flanked by a N-terminal head domain and a non-α-helical C-terminal domain. NFL subunits constitute the core of the filament on which NFM and NFH are anchored through coiled-coil interactions. The C-terminal domains of NFM and NFH form side arms that project from the filament [2]. These domains have been shown to be involved in the rate of NF transport, the radial growth of axons and the regulation of inter-filament affinity [3]–[8]. They also bind microtubules (MTs), either directly [9]–[11] or using Microtubule-Associated Proteins (MAPs) as linkers between NFs and MTs [12]. The association with MTs depends on the phosphorylation state of the C-terminal domain: dephosphorylation of this domain by alkaline phosphatase promotes the NF/MT interaction while phosphorylation by the tau protein kinase II dissociates NFs from MTs.

We recently showed that NFs also contain sites in their N-terminal domains able to bind un-polymerized tubulin. Peptides corresponding to these motifs (named TBS for **T**ubulin-**B**inding **S**ites) inhibit the *in vitro* polymerization of MT. Moreover, a 24-amino acid (AA) peptide corresponding to the second tubulin-binding site located on the NFL subunit (NFL-TBS.40-63) penetrates selectively in T98G human glioblastoma cells where it destroys the MT network and consequently inhibits cell viability [13], [14]. Considering these properties, this peptide represents an interesting candidate for malignant glioma treatment.

Here, we further analyzed the structure-function of NFL-TBS.40-63 using an alanine scan strategy to clarify the cell entering mechanism. The peptides in which each AA was sequentially replaced by alanine were tested for their ability to penetrate into T98G human glioblastoma cells and to disrupt the MT cytoskeleton. In order to detect possible changes in secondary structure the different peptides were also analyzed by circular dichroism (CD) spectroscopy. Finally, since the NFL-TBS.40-63 peptide contains two phosphorylation sites at serines 17 and 19 (which correspond to serines 55 or 57 of the NFL sequence), and considering the effects of the phosphorylation status of these sites on the axonal transport [15], we investigated the properties of modified NFL-TBS.40-63 containing phosphorylated-serine 17 or 19, or aspartic acid substitutions of serines 17 or 19 to mimic permanent phosphorylation.

## Results and Discussion

**Figure 1:**
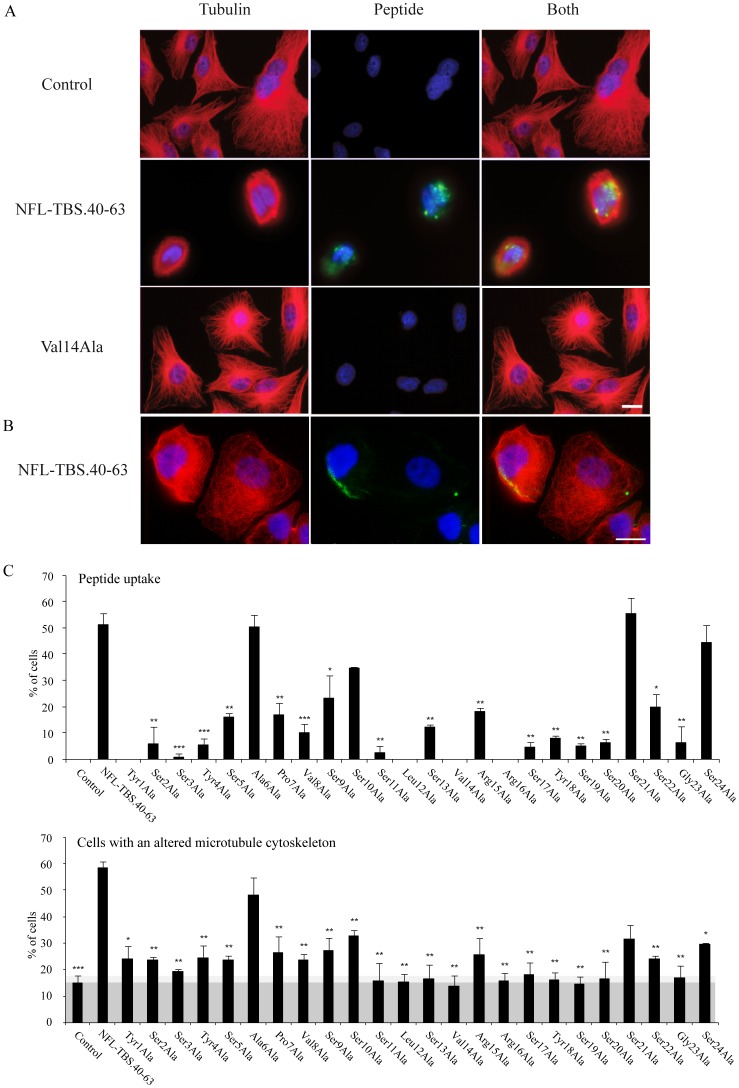
Internalization and effects on glioblastoma T98G cells of the wild type peptide NFL-TBS.40-63 and peptides from the alanine scan. (A): Human glioblastoma T98G cells were incubated in the presence of different peptides at 10 µM during 6 hours. MTs were detected by immunostaining using an anti-tubulin antibody (red), and biotinylated peptides were detected using Alexa-labeled avidin (green). While the original NFL-TBS.40-63 peptide is able to penetrate in these cells, the replacement of valine-14 by alanine typically abolished this property. *White bars*, *20 µm*. (B) Human adrenal carcinoma SW13 cells were incubated in the presence of NFL-TBS.40-63 peptide (10 µM, 6 hours). MTs appear in red, peptide in green, nuclei in blue. *White bars*, *20 µm*. (C): We quantified by microscopy the percentage of T98G cells containing the peptide and those with a destroyed MT network. Experiments were triplicated and a minimum of 200 cells was examined in each experiment. Data are presented as mean and S.E.M. (bars). Asterisks indicate significant level versus control: * p<0.05; ** p<0.005; *** p<0.001. As glioblastoma cells are known to have multiple mutations and abnormalities, the shaded area corresponds to the percentage of cells where MTs are disorganized even in the absence of peptide.

We previously reported the specific uptake of NFL-TBS.40-63 peptide by T98G human glioblastoma cells and its effects on their MT network, while it has no major effect on astrocytes, neurons and other cell types like Hela cells, prostate carcinoma cells, or mouse immortalized fibroblast 3T3 cells [13], [14]. As illustrated in **Figure 1A** the NFL-TBS.40-63 peptide typically enters in glioblastoma cells and disturbs the MT network. It also enters in SW13 cells without affecting the MT network (**Figure 1B**). As SW13 cells do not contain intermediate filaments, these results, as well as those previously published [13], [14], suggest that intermediate filaments (IF) are not directly involved in the activities of the NFL-TBS.40-63 peptide. Here, we further investigated the structure-activity of this peptide to understand its capacity to penetrate in glioblastoma cells and to alter their MT cytoskeleton.

### Revealing the importance of each amino acid by the alanine scanning strategy

In order to evaluate the role of each AA of the NFL-TBS.40-63 peptide in its incorporation into glioblastoma cells and its anti-microtubule activity, we sequentially replaced each AA by alanine, a small and neutral AA (**Table 1**), and human glioblastoma T98G cells were incubated with 10 µM of peptide during 6 hours. Cells containing the biotinylated peptide were detected using Alexa-labeled avidin (green fluorescence) and those displaying a normal or a destroyed MT network were detected with an anti-tubulin antibody (red fluorescence) (**Figure 1A–B**). Then, we counted a minimum of 200 cells per slide containing or not the peptide, and with a normal or destroyed microtubule network (**Figure 1C**). As illustrated on **Figure 1A** glioblastoma cells contain a rich MT network. The normal NFL-TBS.40-63 enters in these cells where it destroys the MT network. On the opposite, the peptide with a Val14Ala replacement fails to enter in cells and does not affect the MT network. The NFL-TBS.40-63 peptide also enters in SW13 cells, which do not have intermediate filaments, but does not affect their MT network (**Figure 1B**).

**Table 1:** Sequences of the peptides used in the Ala-scanning investigation and the modified peptides.

Peptide	Sequence	Molecular weight (g/mol)	PI	Average hydrophilicity	Peptide incorporation (% of cells)	Disintegration of MT (% of cells)
NFL-TBS.40-63	Biotin-YSSYSAPVSSSLSVRRSYSSSSGS-CONH_2_	2712.4	10.3	−0.1	51.2±4.2	58.4±2.3
***Alanine scan***						
Tyr1Ala	Biotin- **A**SSYSAPVSSSLSVRRSYSSSSGS-CONH_2_	2620.1	11.2	0	0	24.1±4.6
Ser2Ala	Biotin-Y**A**SYSAPVSSSLSVRRSYSSSSGS-CONH_2_	2696.4	10.3	−0.1	5.9±6.4	23.5±1.1
Ser3Ala	Biotin-YS**A**YSAPVSSSLSVRRSYSSSSGS-CONH_2_	2696.4	10.3	−0.1	0.8±1.2	19.1±0.9
Tyr4Ala	Biotin-YSS**A**SAPVSSSLSVRRSYSSSSGS-CONH_2_	2620.4	11.2	0	5.3±2.4	24.4±4.6
Ser5Ala	Biotin-YSSY**A**APVSSSLSVRRSYSSSSGS-CONH_2_	2696.4	10.3	−0.1	16.0±1.4	23.6±1.6
Ala6Ala	Biotin-YSSYS**A**PVSSSLSVRRSYSSSSGS-CONH_2_	2712.4	10.3	−0.1	50.3±4.6	48.1±6.6
Pro7Ala	Biotin-YSSYSA**A**VSSSLSVRRSYSSSSGS-CONH_2_	2686.4	10.3	−0.1	16.7±4.6	26.6±5.9
Val8Ala	Biotin-YSSYSAP**A**SSSLSVRRSYSSSSGS-CONH_2_	2684.4	10.3	−0.1	9.9±3.4	23.6±2.1
Ser9Ala	Biotin-YSSYSAPV**A**SSLSVRRSYSSSSGS-CONH_2_	2696.4	10.3	−0.1	23.3±8.4	27.1±4.8
Ser10Ala	Biotin-YSSYSAPVS**A**SLSVRRSYSSSSGS-CONH_2_	2696.4	10.3	−0.1	34.5±0	32.9±1.9
Ser11Ala	Biotin-YSSYSAPVSS**A**LSVRRSYSSSSGS-CONH_2_	2696.4	10.3	−0.1	2.5±2.5	15.9±6.5
Leu12Ala	Biotin-YSSYSAPVSSS**A**SVRRSYSSSSGS-CONH_2_	2670.4	10.3	0	0	15.5±2.8
Ser13Ala	Biotin-YSSYSAPVSSSL**A**VRRSYSSSSGS-CONH_2_	2696.4	10.3	−0.1	12.2±0.8	16.6±5.2
Val14Ala	Biotin-YSSYSAPVSSSLS**A**RRSYSSSSGS-CONH_2_	2684.4	10.3	−0.1	0	13.8±3.9
Arg15Ala	Biotin-YSSYSAPVSSSLSV**A**RSYSSSSGS-CONH_2_	2627.3	9.7	−0.2	18.1±1.3	25.5±6.3
Arg16Ala	Biotin-YSSYSAPVSSSLSVR**A**SYSSSSGS-CONH_2_	2627.3	9.7	−0.2	0	15.6±3.0
Ser17Ala	Biotin-YSSYSAPVSSSLSVRR**A**YSSSSGS-CONH_2_	2696.4	10.3	−0.1	4.6±1.8	18.1±4.4
Tyr18Ala	Biotin-YSSYSAPVSSSLSVRRS**A**SSSSGS-CONH_2_	2620.4	11.2	0	7.9±0.9	16.0±2.8
Ser19Ala	Biotin-YSSYSAPVSSSLSVRRSY**A**SSSGS-CONH_2_	2696.4	10.3	−0.1	5.2±0.7	14.5±2.7
Ser20Ala	Biotin-YSSYSAPVSSSLSVRRSYS**A**SSGS-CONH_2_	2696.4	10.3	−0.1	6.4±1.2	16.4±6.4
Ser21Ala	Biotin-YSSYSAPVSSSLSVRRSYSS**A**SGS-CONH_2_	2696.4	10.3	−0.1	55.4±5.9	31.6±5.1
Ser22Ala	Biotin-YSSYSAPVSSSLSVRRSYSSS**A**GS-CONH_2_	2796.4	10.3	−0.1	19.7±5.0	24.1±1.0
Gly23Ala	Biotin-YSSYSAPVSSSLSVRRSYSSSS**A**S-CONH_2_	2726.4	10.3	−0.1	6.2±6.2	16.9±4.5
Ser24Ala	Biotin-YSSYSAPVSSSLSVRRSYSSSSG**A** -CONH_2_	2696.4	10.3	−0.1	44.3±6.7	29.4±0.6
***Substitutions with functionally equivalent amino acids***						
Tyr1Phe	Biotin- **F**SSYSAPVSSSLSVRRSYSSSSGS-CONH_2_	2696.4	11.2	−0.1	56.3±2.8	41.2±4.0
Leu12Val	Biotin-YSSYSAPVSSS**V**SVRRSYSSSSGS-CONH_2_	2698.4	10.3	−0.1	58.6±5.0	43.8±1.7
Val14Leu	Biotin-YSSYSAPVSSSLS**L**RRSYSSSSGS-CONH_2_	2726.4	10.3	−0.1	44.2±2.2	39.2±2.4
Arg16Lys	Biotin-YSSYSAPVSSSLSVR**K**SYSSSSGS-CONH_2_	2684.4	10.1	−0.1	32.9±1.3	36.9±0.9

A change in activity due to the replacement of an AA may reflect the loss of a specific contact with an interacting molecule [16], like a protein or a lipid molecule, or it can be due to structural modifications of the peptide resulting from the sequence modification [17]. It should be noted that small peptides like NFL-TBS.40-63 generally exist in solution as a mixture of different conformations or sub-states, whose population can vary with changes in the environment or limited sequence modification.

From immuno-cytochemical results and their quantification (**Figure 1A and C**), it appears that most of the AA substitutions by alanine reduced the cell penetration activity of the peptide, except for Ser21Ala and Ser24Ala. Moreover, most substitutions of the AA with alanine decreased the capacity of the peptide to alter the MT network when compared to the original NFL-TBS.40-63 peptide (**Figure 1C**, **Table 1**). However, replacement of Ser21 by alanine did not affect the uptake of the peptide but reduced its capacity to alter the MT network.

**Figure 2:**
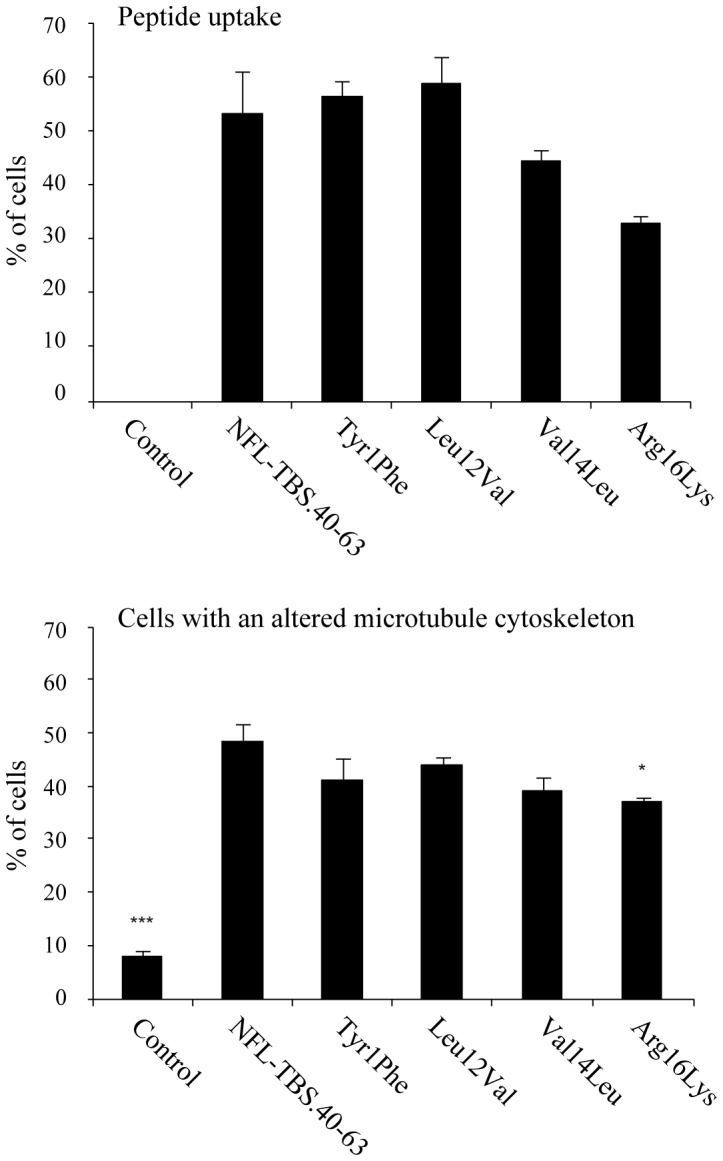
Replacement of AA residues with functionally equivalent residues restores the properties of NFL-TBS.40-63. Using a similar experimental approach to that described in Figure 1, the amino-acids at position 1, 12, 14 and 16 were replaced by amino-acids with similar properties. While substitution of these AAs by alanine abolished their properties, replacement of these AAs by functionally equivalent residues restore the capacity to penetrate in cells and to alter the MT cytoskeleton. As in Figure 1A, experiments were triplicated and a minimum of 200 cells was analyzed in each experiment.

Alanine substitutions at position 1, 12, 14 and 16 abolished the penetration of the peptide into cells. To confirm the crucial role played by these AA, they were replaced by functionally equivalent AA (Tyr1Phe, Leu12Val, Val14Leu and Arg16Lys) (**Figure 2**, **Table 1**). With the exception of Arg16Lys, which displayed a lower activity, such substitutions restored the wild type activity of the peptide, confirming the crucial role of these residues.

Internalization of the NFL-TBS.40-63 peptide by glioblastoma cells may result from either its intrinsic capacity to cross the cell membrane through endocytosis, or its capacity to interact with a cell receptor. In the first case, the peptide would belong to the class of cell penetrating peptides (CPP). These peptides have been investigated for the last ten years for their high potential in cell delivery engineering, especially with a therapeutic objective. Cell penetrating peptides are generally defined as short (less than 30 AA) cationic peptides, which are able to penetrate cell membranes [18]. In general, CPP uptake involves an initial rapid electrostatic interaction of positively charged residues with the plasma membrane before their intracellular delivery, but the exact mechanism is still unclear [19]. Notably, the positive charge itself is not sufficient for internalization. Futaki et al. showed that an octamer of arginine (Arg)_8_ was successfully internalized, whereas (Arg)_16_ had no translocation property [20].

The NFL-TBS.40-63 peptide displays some of the CPP characteristics including its short sequence (24 AA) and its net positive charge at physiological pH (PI = 10.3). However, contrary to the known CPPs, which are composed mostly of arginines and lysines, the NFL-TBS.40-63 peptide contains only 2 arginine residues and no lysine. In addition, we showed previously that scrambled peptides (including NFL-SCR), or a peptide with the same AA composition but in a reversed sequence (NFL-SBT), do not penetrate in cells (**Table 2**). As such peptides have similar positive charge, these results indicate that the positive charge of the NFL-TBS.40-63 sequence alone is not sufficient to account for its cell-penetrating ability [13]. Moreover, the exact sequence is crucial to preserve the properties of this peptide, which may reveal a preferred and specific conformation.

**Table 2:** Sequences of reverse (NFL-SBT) and scrambled (NFL-SCR) peptides, as well as the phosphorylated peptides.

Peptide	Sequence	Molecular weight (g/mol)	PI	Average hydrophilicity	Peptide incorporation (% of cells)	Disintegration of MT (% of cells)
NFL-TBS.40-63	Biotin-YSSYSAPVSSSLSVRRSYSSSSGS-CONH_2_	2712.4	10.3	−0.1	51.2±4.2	58.4±2.3
**Scrambled peptides**						
NFL-SBT	Biotin-SGSSSSYSRRVSLSSSVPASYSSY-CONH_2_	2712.4	10.3	−0.1	0	32.41±0.87
NFL-SCR	Biotin-SLGSPSSSVRASYSSSRSYVYSSS-CONH_2_	2712.4	10.3	−0.1	64.2±4.2	18.18±3.2
***Phosphorylated and acid aspartic peptides***						
Ser17SerP	Biotin-YSSYSAPVSSSLSVRR**Sp**YSSSSGS-CONH_2_	2792.4	10.3	−0.1	32.8±8.7	22.9±1.3
Ser19SerP	Biotin-YSSYSAPVSSSLSVRRSY**Sp**SSSGS-CONH_2_	2792.4	10.3	−0.1	27.3±12.1	19.4±2.1
Ser17,19SerP	Biotin-YSSYSAPVSSSLSVRR**Sp**Y**Sp**SSSGS-CONH_2_	2872.4	10.3	−0.1	32±2.1	11.0±3.0
Ser17Asp	Biotin-YSSYSAPVSSSLSVRR**D**YSSSSGS-CONH_2_	2740.4	9.7	0	31.3±11.2	22.2±3.4
Ser19Asp	Biotin-YSSYSAPVSSSLSVRRSY**D**SSSGS-CONH_2_	2740.4	9.7	0	25.2±4.8	19.2±1.5
Ser17,19Asp	Biotin-YSSYSAPVSSSLSVRR**D**Y**D**SSSGS-CONH_2_	2768.4	6.8	0.1	29.0±12.7	18.6±6.8

### The properties of the NFL-TBS.40-63 peptide are affected by their phosphorylation status

Neurofilaments are known phospho-proteins, and multiple aspects of their biology, including assembly and axonal transport, are regulated by their phosphorylation status. In particular, aberrant NF phosphorylation is a pathological hallmark of several human neurodegenerative disorders [21]. Phosphorylation sites located along the N-terminal domains, especially those of NFL and NFM, are essential for their assembly, their axonal transport or their interactions with MTs [22]–[30]. In particular, modifications of serines 2, 55 and 57 of NFL (i.e. replacement of serine by aspartic acid) alter the axonal transport of NFs [15]. As they are present in the NFL-TBS.40-63 peptide sequence (serine 17 and 19), we analyzed the possible consequence of the phosphorylation of these AA on the internalization of the peptide into glioma cells and on the anti-microtubular activity. Replacement of serines 17 and 19 with aspartic acid to mimic permanent phosphorylation was also examined.

**Figure 3:**
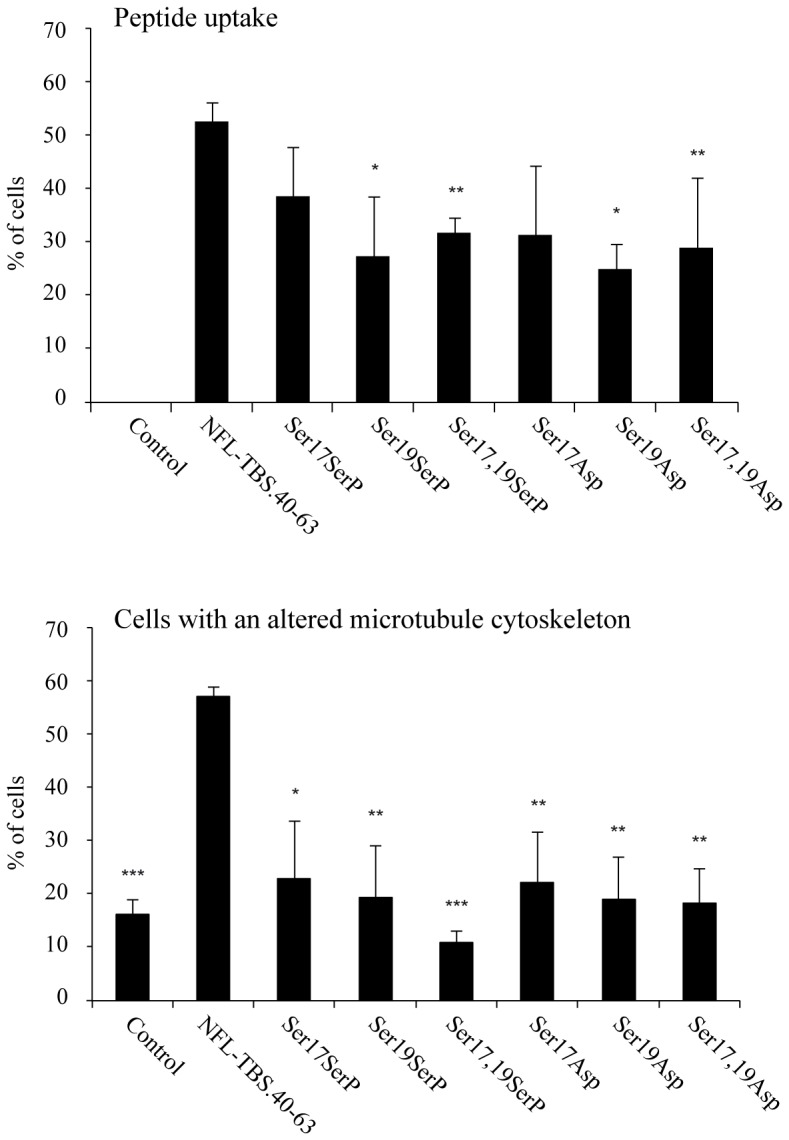
Phosphorylation of NFL-TBS.40-63 affects its properties. Replacement of Ser-17 or Ser-19 by a chemically phosphorylated serine, or by Asp strongly affected the capacity of the peptide to penetrate in cells and to destroy the MT network. The experimental conditions are similar to those described in Figure 1A.

Immunocytochemical studies revealed that all the phosphorylated peptides (Ser17SerP, Ser19SerP, Ser17,19SerP), as well as peptides in which Ser was replaced with Asp (Ser17Asp, Ser19Asp, Ser17,19Asp), showed a decreased internalization, when compared to the original NFL-TBS.40-63 peptide (**Figure 3**, **Table 2**). Moreover, the MT network integrity was not altered when glioma cells were treated with these peptides. These results indicate that the phosphorylation status of serine 17 and 19 is crucial for both the internalization and the effects of the NFL-TBS.40-63 peptide on glioma cells. Phosphorylation clearly opposes these effects.

### Analysis of the folding and stability of the NFL-TBS.40-63 peptide by circular dichroism

To investigate whether the alteration of the activity of the peptide following changes of its sequence or its phosphorylation level may be correlated to a change in its folding and stability, we analyzed the structure of peptides by circular dichroism (CD) measurements. The CD signal in the far UV region (below 250 nm) is related to the secondary structure of proteins [31], [32]. The CD spectra are usually measured in three solvents: (1) 10 mM potassium phosphate with 200 mM KF, pH 7.4, (2) 2 mM Sodium Dodecyl Sulfate (SDS) and (3) pure water. KF/phosphate buffer is similar in ionic strength and pH to the buffer used for cell treatment with peptide. The actual cell treatment buffer cannot be used for the CD measurements because of its high UV absorption in the far UV region. SDS mimics hydrophobic environment at low concentrations (2 mM) where micelle formation should not occur [33]. The peptide structure in the presence of SDS could thus indicate a potential conformation of the peptide when in contact with the membrane or the partner protein. The CD was also measured in pure water as a reference to evaluate the effect of buffer and SDS on the peptide conformation. All the CD measurements were performed on the peptides that were used for the biological tests.

**Figure 4:**
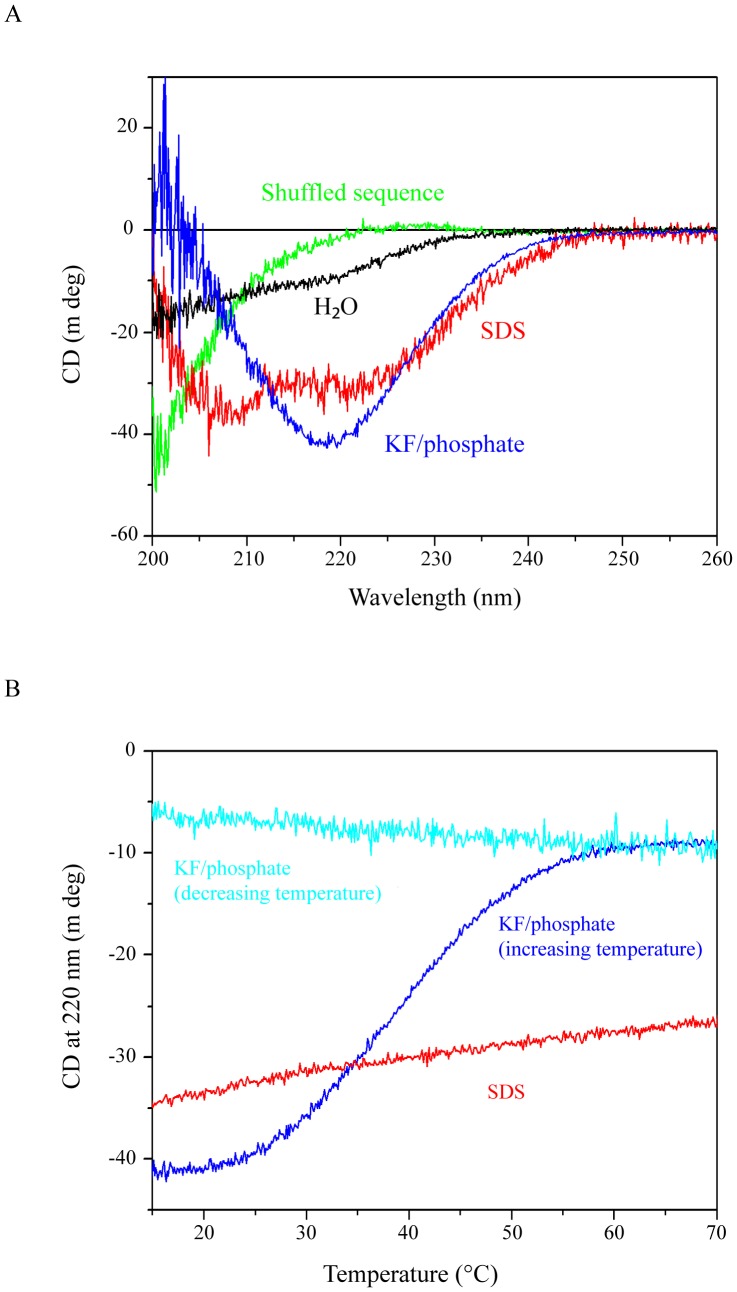
CD spectra of NFL-TBS.40-63 peptide. (A): CD spectra of 20 µM biotinylated NFL-TBS.40-63 peptide were measured in pure water (black line), 2 mM SDS (red line) and KF/phosphate buffer (blue line). The spectrum of peptide with a shuffled sequence (green) is also shown. (B): Change in CD signal at 220 nm of 20 µM biotinylated NFL-TBS.40-63 in KF/phosphate buffer (blue line) and 2 mM SDS (red line) with increasing temperature was measured and is presented as a function of temperature. The change with decreasing temperature measured in KF/phosphate buffer (light blue line) is also shown.

The CD spectrum of the original NFL-TBS.40-63 peptide (wt NFL-TBS.40-63) in pure water indicates rather disordered conformation (**black line in** **Figure 4A**): the signal at 200 nm was negative while the ordered conformations (both α-helix and β-sheet) exhibit a positive CD signal at this UV region [31], [32]. However, an additional negative feature could be seen around 220 nm, suggesting partial folding into α-helix or β-sheet, because an α-helix structure exhibits a negative band at 222 nm and β-sheet at 218 nm, while disordered peptides usually exhibit no or a positive signal around 220 nm. In fact, when the CD spectrum was analyzed by using Jasco secondary estimation software with Reed's reference set as reference spectra, the secondary structure was estimated to be composed of 25% β-sheet and 75% random coil. This differs from the CD spectra of a peptide with the same AA composition but in a shuffled sequence (NFL-SCR) (**green line in** **Figure 4A**) or a reverse sequence (NFL-SBT) (data not shown): the signal above 220 nm is positive in their CD spectra. These peptides exhibited typical CD spectra corresponding to a completely disordered structure, while the wild-type NFL-TBS.40-63 peptide has some tendency to be folded in a particular conformation.

Furthermore, the CD spectrum of wild-type NFL-TBS.40-63 peptide was modified by addition of 2 mM SDS (**blue line in** **Figure 4A**), which creates a hydrophobic environment and may mimic the situation around the membrane, in a way indicating more ordered structures: the signal at 200 nm became positive and the negative band around 220 nm displayed an higher intensity. Two negative bands (one centered at 222 nm and the other at 209 nm) suggest the presence of α-helical structures. The spectral analysis with Reed's reference set predicts 20% α-helix, 40% β-sheet and 40% random coil. Thus, the peptide may be folded partially in an α-helical structure when it interacts with the membrane. However, addition of any mild detergents (IGEPAL CA-630, Nonidet P40, Tween 20, Triton X-100) to the peptide in pure water did not affect the CD signal.

In KF/phosphate buffer, which mimics the cell treating buffer, the peptide exhibited another type of CD spectrum: the signal at 200 nm is positive and the negative band around 220 nm was greater in intensity compared to that in pure water (**blue line in** **Figure 4A**). Since the negative band was centered at 218 nm instead of 222 nm and there was no clear negative band at 209 nm, another typical negative band for an α-helical structure, the peptide is probably folded in an antiparallel β-sheet structure. The spectral analysis with Reed's reference set predicts 7% α-helix, 56% β-sheet and 36% random coil.

The different structural characteristics observed in KF/phosphate buffer or in SDS correspond to different properties in terms of stability. In KF/phosphate buffer, the peptide was not stable when the temperature was increased: thermal unfolding occurred with Tm of about 40°C (**blue line in** **Figure 4B**). In addition, no refolding was observed when the temperature was lowered from 70 to 10°C at a rate of 1°C per min and even after 12 hours incubation at 20°C (**light blue line in** **Figure 4B**). Corresponding to this structural feature, the peptide once heated could not penetrate into the cells. In contrast, the peptide in 2 mM SDS was stable: no large signal change in CD spectrum was observed when the temperature was increased up to 70°C (**red line in** **Figure 4B**).

**Figure 5:**
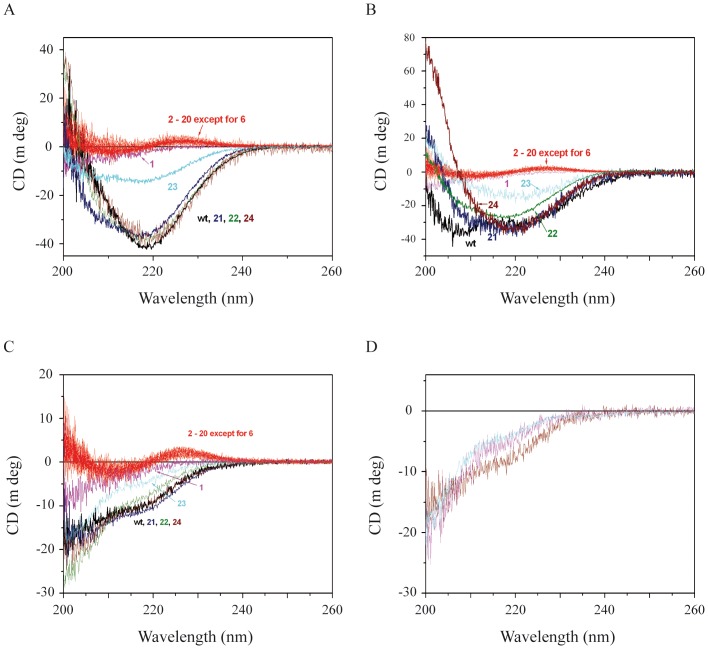
CD spectra of wt and alanine-substituted peptides. CD spectra of 20 µM biotinylated wt and alanine-substituted NFL-TBS.40-63 peptides were measured in KF/phosphate buffer (Panel A), 2 mM SDS (Panel B) and pure water (Panel C). The spectra of wt peptide are in black. The spectra of peptides with a substitution by alanine at positions from 2 to 20 (except at position 6 where the original AA is alanine) are similar and represented by red lines. The spectra of wild-type (black), Tyr1Ala (magenta), Ser21Ala (dark blue), Ser22Ala (olive), Gly23Ala (light blue) and Ser24Ala (wine) are also presented. The number corresponds to the position of the replaced AA residue. CD spectra of 20 µM NFL-TBS.40-63 peptides phosphorylated at positions 17 (wine), 19 (magenta) and the two positions (cyan) were measured in KF/phosphate buffer (Panel D).

The dependency on the environment of the NFL-TBS.40-63 peptide structure is characteristic of its exact sequence arrangement. No modification in the CD signal of its shuffled (NFL-SCR) or reverse sequence peptide (NFL-SBT) could be observed following addition of SDS or in KF/phosphate buffer (data not shown). Moreover, both the structural characteristics of the peptide and its dependency on the environment can be greatly modified by punctual substitutions. As described below, the substitution of only one AA with alanine at certain positions of the peptide affected the CD signal, indicating major change in the structure (**Figure 5**).

In summary, the CD analysis indicates that the NFL-TBS.40-63 peptide folds into secondary structure elements and exhibits both α-helix and β-sheet structures, with a more pronounced importance of the α-helical character in a hydrophobic medium relative to a polar environment. Such data argue that for this peptide, membrane penetration would necessitate the formation of specific secondary structures. However, this does not imply that the peptide necessarily acts as a CPP, as the relation between the properties of CPP and their structural folding is not straightforward. While an α-helical structure is generally found or predicted for these peptides, β-sheet structures have also been described for some CPPs [34]. Furthermore, the α-helical structure of antennapedia can adopt a β-sheet structure in the presence of a charged lipid monolayer [35].

To further evaluate the importance of the structure/function relationships we also analyzed by CD the folding and stability of the peptide following alanine substition of each AA, or their phosphorylation state.

### Alanine substitutions and phosphorylation affect the folding of NFL-TBS.40-63 peptide

The CD spectra of alanine-substituted peptides can be separated in two classes (**Figure 5**). The spectra of peptides in which an AA residue at C-terminal part (21 to 24) was substituted with alanine exhibited a large negative band around 218 nm in the presence of SDS or in the KF/phosphate buffer. The CD spectra of these peptides depended upon the solvent just like the spectrum of the wild-type peptide. The peptides appeared rather disordered in pure water, in mainly anti-parallel β-sheet in KF/phosphate buffer and in a mixture of β-sheet and α-helical structures in 2 mM SDS. There was some correlation between the CD spectrum in KF/phosphate buffer and cell penetrating capacity. Gly23Ala, which exhibited only weak cell-penetrating activity, also displayed a CD signal corresponding to less ordered structure (**light blue line in** **Figure 5A**).

The CD spectra of peptides in which a residue is substituted with alanine at positions 2 to 20 (except for position 6, which is originally alanine) exhibited much weaker CD signals (**red lines in** **Figures 5A, B and C**). However, the spectra had a well-defined shape: a positive band centered at 228 nm, a negative band at 210 nm and a positive band at 200 nm. This kind of CD spectrum is reported to correspond to another type of β-sheet structure according to Yang et al. [36]. We could not fit the spectra using Reed's reference set, but obtained a reasonable fit using Yang's reference: 6% α-helix, 49% β-sheet, 28% β-turn and 16% random coil. The spectra were not affected by addition of SDS or in KF/phosphate buffer condition. The structure thus appeared very stable. Even increasing the temperature up to 80°C did not significantly affect these CD spectra (data not shown). Almost all peptides exhibiting such CD spectra did not show any cell-penetrating capacity except Ser9Ala and Ser10Ala. These two particular peptides penetrated in cells significantly although to a much lesser extent than the wt peptide. The peptide in which the residue at position 1 was substituted with alanine exhibited CD signals corresponding to a random coil structure in all solvents, as well as a loss of biological activities. Thus, tyrosine at position 1 is important for the folding of this peptide and its activities.

As exposed in the previous section, we observed that the substitution by alanine at positions 1, 12, 14 or 16, strongly perturbed the peptide activity, but the substitution with physico-chemically similar AA (Tyr1Phe, Leu12Val Val14Leu and Arg16Lys) conserved the activity except for Arg16Lys. We therefore examined the CD of these peptides. The CD spectra of peptides containing Tyr1Phe, Leu12Val or Arg16Lys substitution exhibited a CD signal similar to the wt peptide in KF/phosphate buffer. The CD spectrum of the peptide containing Val14Leu substitution corresponded to a less ordered structure than the wt peptide, but clearly different from the peptide with Val14Ala substitution. These results indicate that the physico-chemical properties of these particular AA are important for the folding of the peptide in the active form. Moreover, the fact that the peptide with Arg16Lys substitution folds in a similar way to wt peptide but exhibits lower activity indicates the importance of this residue for the interaction with other proteins or with the membrane.

CD measurements showed that phosphorylation of the peptide at positions 17 and 19 destabilized the β-sheet structure (**Figure 5D**), in correlation with the decrease in cell-penetrating capacity. The CD analysis estimates that the phosphorylation at position 17 (**wine line in** **Figure 5D**) decreased the β-sheet content to 40% (from 56% content in the wt peptide) and increased the random coil content to 53% (36% in the wt peptide), while that at position 19 (**magenta line in** **Figure 5D**) decreased the β-sheet content to 34% and increased the random coil content to 60%. The phosphorylation of both sites (**cyan line in** **Figure 5D**) produced changes almost similar to those produced by single phosphorylation at position 17. Again, there is a correlation between the activity and the structure of peptide.

Together, these results reveal the importance of folding for the activity: both the peptides with scrambled or reversed sequence, as well as the peptide in which tyrosine 1 is replaced by alanine, do not cross the cell membranes and are not structured. In addition, the cell penetrating activity appears to correlate with a particular structural pattern. Moreover, all the mutated peptides, which exhibit a CD spectrum analogous to that of the wild type peptide (substitution with alanine at positions 21, 22, and 24 or functionally equivalent Tyr1Phe, Leu12Val and Arg16Lys substitutions) show a notable cell penetration activity. This was generally not the case for mutations that led to an ostensibly different CD spectrum, corresponding to different structural characteristics. These results, together with the absence of internalization of NFL-TBS.40-63 peptide composed of D-amino acids, and the saturating process of internalization in glioma cells demonstrated by FACS [13], suggest that a receptor may participate to its internalization. In that case, differences in uptake patterns of the peptide between glioma cells and normal cells could be due to differences of its interaction with a particular cell surface receptor expressed specifically in glioma. Our observations leave open the two possible options for crossing the glioblastome membrane, either directly via endocytosis or through its interaction with a cell receptor.

Another interesting feature of the NFL-TBS.40-63 peptide is its sensitivity to the environment (KF/phosphate buffer or SDS), and its remarkable stabilization by the presence of SDS. Such a characteristic is also observed for mutated forms of the NFL-TBS.40-63 peptide that still penetrate in cells. Moreover, the results suggest that Tyr1, Leu12 and Ser21 interact with tubulin as their tubulin binding activity decreases upon replacement by alanine or by residues with similar chemical properties, while the cell penetration is not compromised and the CD spectra are similar. These data suggest that a different conformation is required for anti-MT activity than for crossing the membrane. The alternative fold could be stabilized by tubulin binding.

It is noteworthy that the structure of the peptide is stabilized in the KF/phosphate buffer or in the presence of SDS. This indicates that some hydrophobic contacts in the peptide are important for the folding. The fact that the replacement of residues in the C-terminal part with alanine residue, which has distinct volume occupancy, destabilizes the structure suggests that this part is involved in the folding. On the other hand, replacement of residues in the N-terminal half by alanine promotes another folding.

### Molecular modeling of NFL-TBS.40-63 peptide

**Figure 6:**
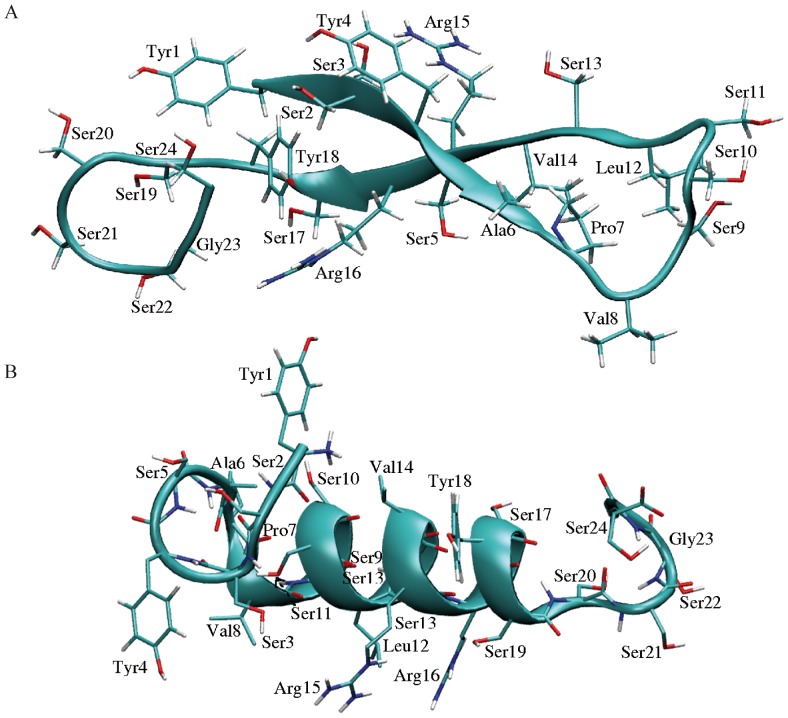
Predicted fold of NFL-TBS.40-63 peptide. A. The 3D structure of NFL-TBS.40-63 predicted by PEP-FOLD shows a β-hairpin involving the N-terminal section (1–5) and a central section (14–18) of the peptide. The C-terminal end shows the initiation of an α-helix. B. Alternative 3D structure predicted by PEP-FOLD, where the peptide mainly presents an α-helix fold. The figures were prepared using VMD [49].

Two extreme folding geometries were predicted by the PEP-FOLD program for the NFL-TBS.40-63 peptide (**Figure 6**). In the first, the peptide is predominantly folded into a β-hairpin geometry extended over five consecutive amino acids (residues 1 to 5 are connected to residues 18 to 14 respectively), with a short C-terminal section presenting an α-helix character (**Figure 6A**). The second geometry is characterized by a predominant α-helix folding (**Figure 6B**). The two geometries present almost equivalent internal energies, with respectively values of −25.0 and −26.0 kcal.mol^−1^. Each of them conserved its structural characteristics, in terms of secondary structure, over 2 ns of molecular dynamics simulation in explicit solvent. The structure with dominant α-helix character showed transient instabilities of one of its four helix turns situated at the N-ter side during 300 ps, after which the helix turn recovered stable interactions. Shifts in the β-strand pairing were also observed after 1 ns, indicating some structural variability. These results show that the two structures represent conformational substates with medium to low stability, which may coexist in solution. The mutations of residues Tyr4, Ser9, Ser11, Leu12, Ser13, Val14, Arg15, Ser17, and Ser19 into alanine were found to destabilize the β-hairpin part of the peptide and stabilize an α-helix conformation.

In conclusion, the combination of our immuno-cytochemical and structural analyses strongly suggests that the NFL-TBS.40-63 peptide presents specific folds when crossing the membrane as well as when it interacts with tubulin, but that these structures are not necessarily identical. As these conformations are affected each time that the properties to penetrate or to perturb the MT network are also affected, these results indicate a good structure/function relationships. Moreover, the peptide's ability to modify its structure in different conditions such as the cell interior versus hydrophobic environment appears a hallmark of the peptide activity. This structure/function relationship suggests importance of structure, not simple amino acid composition, for the activities of the peptide, including the selectivity to glioblastoma cells. Further studies will aim at characterizing the conformational equilibrium of the NFL-TBS.40-63 peptide in different conditions (temperature, solvent composition) and its structure when interacting with membranes or tubulin, using theoretical tools like molecular dynamics simulations and experimental structure determination.

## Materials and Methods

### Synthetic peptides

Biotinylated NFL-TBS.40-63 peptide corresponding to the tubulin-binding site located on NFL and biotinylated analogues (Tables 1 and 2) were synthesized at more than 95% purity by Millegen (Toulouse, France). Peptides were dissolved in distilled water at a concentration of 1 mM.

### Cell culture

Human glioblastoma cell line T98G was obtained from ATCC (Manassas, VA, USA). Human adrenal carcinoma cell line SW13 was kindly provided by Zhigang XUE (UPMC, Paris). Cells were grown in DMEM (Dulbecco's Modified Eagles Medium) media (Lonza France) containing respectively 10% and 2% fetal calf serum (Lonza France), 1% L-glutamine (Lonza France), and 1% penicillin/streptomycin (Sigma) in a humidified incubator gassed with 5% CO_2_ (37°C) until 80–90% confluence was reached.

### Immunocytochemistry

Cells were plated on cover slips and cultured in media containing biotinylated peptides (10 µM) for 6 hours. After being washed in Phosphate Buffered Saline (PBS), the cells were fixed for 10 min in 4% Paraformaldehyde (PFA in PBS), and washed 3 times in PBS. The cells were then incubated during 10 min in a 0.5% triton X-100 permeabilization solution, and washed 3 times in PBS. Next, they were incubated in a blocking solution (PBS 5% BSA) for 15 min, then incubated with mouse anti-ß-tubulin antibody (Sigma) 1/200 overnight. Tubulin and biotinylated peptides were localized using respectively Alexa 568 nm anti-mouse antibody and streptavidin Alexa 488 nm (Molecular Probes) 1/200 for 1 hour, followed by washing in PBS. The preparations were counterstained with 3 µM 4′6-diaminido-2-phenylindole (DAPI; Sigma) for 5 min, washed twice with PBS, then cover slips were mounted with an antifading solution.

Observations were performed with an Olympus confocal microscope (BX50) using Fluoview.3.1. Software or a Leica DMI6000 inverted microscope and analyzed with Metamorph 7.1.7.0. software. We counted cells containing the peptide and cells displaying a destroyed MT network. Experiments were repeated at least three times, and a minimum of 200 cells was scored during each experiment.

### Circular dichroism measurements

Circular dichroism (CD) spectra were measured in a J-810 CD spectrometer (Jasco, Japan) in step mode (bandwidth: 2 nm; interval: 0.1 nm; response time: 0.125 s). The spectra were averaged over 3 scans to increase the signal to noise ratio. To optimize the measurements, a 1 cm×0.2 cm mini-quartz cell with four windows (Hellema, Germany) was used in such a way that the spectra from 260 to 200 nm were measured with a 1 cm pathlength and those between 220 and 185 nm with 0.2 cm pathlength. After normalization for pathlength (1 cm), the superimposition of two separated spectra in the 200–220 nm region was verified and the entire spectra reconstituted. The temperature of measurement was controlled with a Peltier-effect temperature controller, and was usually 20°C. The structural stability of peptides was studied by measuring the variation in CD signal at 220 nm (bandwidth: 10 nm; interval: 1 s; response time: 0.5 s; pathlength: 1 cm) when the temperature was increased by 1°C/min from 20 to 85°C. All the CD experiments were performed with 20 µM peptide under 3 buffer conditions: pure water, 2 mM SDS and 10 mM potassium phosphate pH 7.4 with 200 mM KF.

The secondary structures were estimated from the CD spectra with Jasco secondary structure estimation software. The spectra from 195 to 200 nm were decomposed to α-helix, β-sheet, β-turn and random coil elements using either Reed's [37] or Yang's reference set [38] as reference spectrum of each element as described [37], [38]. We usually obtained better fits using Reed's reference.

### Molecular modeling

Molecular modeling was carried out using the program PEP-FOLD (web server http://bioserv.rpbs.univ-paris-diderot.fr/PEP-FOLD/, [39]). PEP-FOLD predicts “ab initio” the folding characteristics of peptides comprising 9 to 25 amino-acids. It is based on structural alphabet SA letters describing the possible conformation of groups of four consecutive amino acids, which are selected and assembled via a genetic algorithm [40]. Structure reconstruction and energy evaluation rely on the coarse grain force field OPEP [41]. This program has been used successfully by several groups to predict biologically relevant peptide structures [42]–[44]. The sequence of the 24 amino acid peptide NFL-TBS.40-63 and all its alanine mutants were submitted to the PEP-FOLD server. Two protocols were used, either the default protocol or one using the PSI-PRED option which incorporates information from the homology-based PSI-PRED predictor [45]. Note that a recent update of the PEP-Fold program unifies the results of the two options, while supporting up to 35 amino acids.

Each one of the two main structures predicted for the wild type peptide (respectively represented in Figure 6A and Figure 6B) was submitted to a 2 ns, fully solvated, molecular dynamics simulation using the GROMACS software version 4.5.4 [46] and the OPLS/AA-2001 force field [47]. The simulation boxes were filled with 5714 (for the structure in Figure 6A) or 3873 (structure in Figure 6B) SPC/E water molecules. Two chlorure ions were added to ensure electric neutrality. After energy minimization, each structure was submitted to a sequence of two 100 ps equilibration phases, respectively performed under canonical (NVT) and isothermal-isobaric (NPT) ensembles. Production was conducted in the NPT ensemble, using Particle-Mesh-Ewald treatment [48] for long range electrostatics interactions.

### Statistical analysis

Data are presented as mean and Standard Error of the Mean (S.E.M.) (bars). Cell countings were analyzed by Student's *t* test using Prism version 3.00 (GraphPad Software, San Diego, CA). We distinguished three different levels of variation with respect to control: p<0.05; p<0.005; p<0.001 (see Figure 1).

## References

[1] Lee MK, Cleveland DW (1996) Neuronal intermediate filaments. Annual Review of Neuroscience 19: 187–217. 10.1146/annurev.ne.19.030196.001155 8833441

[2] Hirokawa N (1982) Cross-linker system between neurofilaments, microtubules, and membranous organelles in frog axons revealed by the quick-freeze, deep-etching method. The Journal of cell biology 94: 129–142. 6181077 10.1083/jcb.94.1.129 PMC2112203

[3] Carden MJ, Trojanowski JQ, Schlaepfer WW, Lee VM (1987) Two-stage expression of neurofilament polypeptides during rat neurogenesis with early establishment of adult phosphorylation patterns. The Journal of Neuroscience 7: 3489–3504. 3119790 10.1523/JNEUROSCI.07-11-03489.1987 PMC6569049

[4] Eyer J, Leterrier JF (1988) Influence of the phosphorylation state of neurofilament proteins on the interactions between purified filaments in vitro. Biochemical journal 252: 655–660. 2844152 10.1042/bj2520655 PMC1149198

[5] Eyer J, Peterson A (1994) Neurofilament-deficient axons and perikaryal aggregates in viable transgenic mice expressing a neurofilament-beta-galactosidase fusion protein. Neuron 12: 389–405. 8110465 10.1016/0896-6273(94)90280-1

[6] Zhu QZ, Couillard-Despres S, Julien JP (1997) Delayed maturation of regenerating myelinated axons in mice lacking neurofilaments. Experimental Neurology 148: 299–316. 9398473 10.1006/exnr.1997.6654

[7] Ohara O, Gahara Y, Miyake T, Teraoka H, Kitamura T (1993) Neurofilament deficiency in quail caused by nonsense mutation in neurofilament-L gene. Journal of Cell Biology 121: 387–395. 8468353 10.1083/jcb.121.2.387 PMC2200107

[8] Dewaegh SM, Lee VMY, Brady ST (1992) Local modulation of neurofilament phosphorylation, axonal caliber, and slow axonal-transport by myelinating Schwann-cells. Cell 68: 451–463. 1371237 10.1016/0092-8674(92)90183-d

[9] Hisanaga S, Hirokawa N (1990) Dephosphorylation-induced interactions of neurofilaments with microtubules. The Journal of Biological Chemistry 265: 21852–21858. 2254337

[10] Hisanaga S, Kusubata M, Okumura E, Kishimoto T (1991) Phosphorylation of neurofilament H subunit at the tail domain by Cdc2 kinase dissociates the association to microtubules. Journal of Biological Chemistry 266: 21798–21803. 1939202

[11] Miyasaka H, Okabe S, Ishiguro K, Uchida T, Hirokawa N (1993) Interaction of the tail domain of high-molecular-weight subunits of neurofilaments with the COOH-terminal region of tubulin and its regulation by Tau-protein kinase-II. Journal of Biological Chemistry 268: 22695–22702. 8226779

[12] Hirokawa N, Hisanaga S, Shiomura Y (1988) MAP2 is a component of crossbridges between microtubules and neurofilaments in the neuronal cytoskeleton: quick-freeze, deep-etch immunoelectron microscopy and reconstitution studies. The Journal of Neuroscience 8: 2769–2779. 3045269 10.1523/JNEUROSCI.08-08-02769.1988 PMC6569399

[13] Bocquet A, Berges R, Frank R, Robert P, Peterson AC, et al (2009) Neurofilaments Bind Tubulin and Modulate Its Polymerization. Journal of Neuroscience 29: 11043–11054. 19726663 10.1523/JNEUROSCI.1924-09.2009 PMC6665525

[14] Berges R, Balzeau J, Peterson AC, Eyer J (2012) A tubulin binding peptide targets glioma cells disrupting their microtubules, blocking migration, and inducing apoptosis. Molecular Therapy 20: 1367–1377. 22491214 10.1038/mt.2012.45 PMC3392973

[15] Yates DM, Manser C, De Vos KJ, Shaw CE, McLoughlin DM, et al (2009) Neurofilament subunit (NFL) head domain phosphorylation regulates axonal transport of neurofilaments. European Journal of Cell Biology 88: 193–202. 19147253 10.1016/j.ejcb.2008.11.004

[16] Chen J, Serizawa T, Komiyama M (2011) Binding analysis of peptides that recognize preferentially cis-azobenzene groups of synthetic polymers. Journal of Peptide Science 17: 163–168. 21234989 10.1002/psc.1299

[17] Guerrini R, Salvadori S, Rizzi A, Regoli D, Calo G (2010) Neurobiology, Pharmacology, and Medicinal Chemistry of Neuropeptide S and Its Receptor. Medicinal Research Reviews 30: 751–777. 19824051 10.1002/med.20180

[18] Zorko M, Langel U (2005) Cell-penetrating peptides: mechanism and kinetics of cargo delivery. Advanced Drug Delivery Reviews 57: 529–545. 15722162 10.1016/j.addr.2004.10.010

[19] Foerg C, Merkle HP (2008) On the biomedical promise of cell penetrating peptides: Limits versus prospects. Journal of Pharmaceutical Sciences 97: 144–162. 17763452 10.1002/jps.21117

[20] Futaki S, Suzuki T, Ohashi W, Yagami T, Tanaka S, et al (2001) Arginine-rich peptides - An abundant source of membrane-permeable peptides having potential as carriers for intracellular protein delivery. Journal of Biological Chemistry 276: 5836–5840. 11084031 10.1074/jbc.M007540200

[21] Perrot R, Berges R, Bocquet A, Eyer J (2008) Review of the multiple aspects of neurofilament functions, and their possible contribution to neurodegeneration. Molecular Neurobiology 38: 27–65. 18649148 10.1007/s12035-008-8033-0

[22] Julien JP, Mushynski WE (1982) Multiple phosphorylation sites in mammalian neurofilament polypeptides. The Journal of Biological Chemistry 257: 10467–10470. 7202005

[23] Hisanaga S, Gonda Y, Inagaki M, Ikai A, Hirokawa N (1990) Effects of phosphorylation of the neurofilament L protein on filamentous structures. Cell Regulation 1: 237–248. 2100199 10.1091/mbc.1.2.237 PMC361451

[24] Mukai H, Toshimori M, Shibata H, Kitagawa M, Shimakawa M, et al (1996) PKN associates and phosphorylates the head-rod domain of neurofilament protein. Journal of Biological Chemistry 271: 9816–9822. 8621664 10.1074/jbc.271.16.9816

[25] Sihag RK, Jaffe H, Nixon RA, Rong XH (1999) Serine-23 is a major protein kinase A phosphorylation site on the amino-terminal head domain of the middle molecular mass subunit of neurofilament proteins. Journal of Neurochemistry 72: 491–499. 9930720 10.1046/j.1471-4159.1999.0720491.x

[26] Sihag RK, Nixon RA (1991) Identification of Ser-55 as a major protein kinase-A phosphorylation site on the 70-kDa subunit of neurofilaments - Early turnover during axonal-transport. Journal of Biological Chemistry 266: 18861–18867. 1717455

[27] Cleverley KE, Betts JC, Blackstock WP, Gallo JM, Anderton BH (1998) Identification of novel in vitro PKA phosphorylation sites on the low and middle molecular mass neurofilament subunits by mass spectrometry. Biochemistry 37: 3917–3930. 9521713 10.1021/bi9724523

[28] Giasson BI, Mushynski WE (1998) Intermediate filament disassembly in cultured dorsal root ganglion neurons is associated with amino-terminal head domain phosphorylation of specific subunits. Journal of Neurochemistry 70: 1869–1875. 9572270 10.1046/j.1471-4159.1998.70051869.x

[29] Hashimoto R, Nakamura Y, Komai S, Kashiwagi Y, Matsumoto N, et al (2000) Phosphorylation of neurofilament-L during LTD. Neuroreport 11: 2739–2742. 10976954 10.1097/00001756-200008210-00026

[30] Trimpin S, Mixon AE, Stapels MD, Kim MY, Spencer PS, et al (2004) Identification of endogenous phosphorylation sites of bovine medium and low molecular weight neurofilament proteins by tandem mass spectrometry. Biochemistry 43: 2091–2105. 14967049 10.1021/bi030196q

[31] Johnson WC Jr (1990) Protein secondary structure and circular dichroism: a practical guide. Proteins 7: 205–214. 2194218 10.1002/prot.340070302

[32] Norden B, Rodger A, Dafforn T (2010) Linear dichroism and circular dichroism: a textbook on polarized-light spectroscopy. Royal Society of Chemistry (ISBN: 978-1-84755-902-9).

[33] Waterhous DV, Johnson WC (1994) Importance of environment in determining secondary structure in proteins. Biochemistry 33: 2121–2128. 8117668 10.1021/bi00174a019

[34] Oehlke J, Krause E, Wiesner B, Beyermann M, Bienert M (1997) Extensive cellular-uptake into endothelial cells of an amphipathic beta-sheet forming peptide. Febs Letters 415: 196–199. 9350995 10.1016/s0014-5793(97)01123-x

[35] Bellet-Amalric E, Blaudez D, Desbat B, Graner F, Gauthier F, et al (2000) Interaction of the third helix of Antennapedia homeodomain and a phospholipid monolayer, studied by ellipsometry and PM-IRRAS at the air-water interface. Biochimica Et Biophysica Acta-Biomembranes 1467: 131–143. 10.1016/s0005-2736(00)00218-2 10930516

[36] Yang JT, Wu CS, Martinez HM (1986) Calculation of protein conformation from circular dichroism. Methods in enzymology 130: 208–269. 3773734 10.1016/0076-6879(86)30013-2

[37] Reed J, Reed TA (1997) A set of constructed type spectra for the practical estimation of peptide secondary structure from circular dichroism. Analytical Biochemistry 254: 36–40. 9398343 10.1006/abio.1997.2355

[38] Venyaminov SY, Baikalov IA, Shen ZM, Wu CSC, Yang JT (1993) Circular dichroic analysis of denatured proteins - inclusion of denatured proteins in the reference set. Analytical Biochemistry 214: 17–24. 8250221 10.1006/abio.1993.1450

[39] Maupetit J, Derreumaux P, Tuffery P (2009) PEP-FOLD: an online resource for de novo peptide structure prediction. Nucleic Acids Research 37: W498–W503. 19433514 10.1093/nar/gkp323 PMC2703897

[40] Maupetit J, Derreumaux P, Tuffery P (2010) A Fast Method for Large-Scale De Novo Peptide and Miniprotein Structure Prediction. Journal of Computational Chemistry 31: 726–738. 19569182 10.1002/jcc.21365

[41] Maupetit J, Tuffery P, Derreumaux P (2007) A coarse-grained protein force field for folding and structure prediction. Proteins 69: 394–408. 17600832 10.1002/prot.21505

[42] Duvignaud JB, Leclerc D, Gagne SM (2010) Structure and dynamics changes induced by 2,2,2-trifluoro-ethanol (TFE) on the N-terminal half of hepatitis C virus core protein. Biochemistry and Cell Biology-Biochimie Et Biologie Cellulaire 88: 315–323. 20453932 10.1139/o09-155

[43] Kawaguchi A, Suzuki T, Kimura T, Sakai N, Ayabe T, et al (2010) Functional analysis of an alpha-helical antimicrobial peptide derived from a novel mouse defensin-like gene. Biochemical and Biophysical Research Communications 398: 778–784. 20637182 10.1016/j.bbrc.2010.07.028

[44] Steckbeck JD, Craigo JK, Barnes CO, Montelaro RC (2011) Highly Conserved Structural Properties of the C-terminal Tail of HIV-1 gp41 Protein Despite Substantial Sequence Variation among Diverse Clades: Implications for Functions in Viral Replication. Journal of Biological Chemistry 286: 27156–27166. 21659530 10.1074/jbc.M111.258855 PMC3149309

[45] Jones DT (1999) Protein secondary structure prediction based on position-specific scoring matrices. Journal of Molecular Biology 292: 195–202. 10493868 10.1006/jmbi.1999.3091

[46] Hess B, Kutzner C, van der Spoel D, Lindahl E (2008) GROMACS 4: Algorithms for highly efficient, load-balanced, and scalable molecular simulation. Journal of Chemical Theory and Computation 4: 435–447. 26620784 10.1021/ct700301q

[47] Kaminski GA, Friesner RA, Tirado-Rives J, Jorgensen WL (2001) Evaluation and Reparametrization of the OPLS-AA Force Field for Proteins via Comparison with Accurate Quantum Chemical Calculations on Peptides. Journal of Physical Chemistry B 105: 6474–6487.

[48] Essman U, Perela L, Berkowitz ML, Darden T, Lee H, et al (1995) A smooth particle mesh Ewald method. Journal of Chemical Physics 103: 8577–8592.

[49] Humphrey W, Dalke A, Schulten K (1996) VMD: Visual molecular dynamics. Journal of Molecular Graphics & Modelling 14: 33–38. 10.1016/0263-7855(96)00018-5 8744570

